# Tribological Properties of Blocky Composites with Carbon Nanotubes

**DOI:** 10.3390/ijms25073938

**Published:** 2024-04-01

**Authors:** Chaoxiang Hu, Yunqing Gu, Qianfeng Qiu, Hongxin Ding, Jiegang Mou, Denghao Wu, Longbiao Ma, Maosen Xu, Chengqi Mou

**Affiliations:** 1 College of Metrology Measurement and Instrument, China Jiliang University, Hangzhou 310018, China; p22020854038@cjlu.edu.cn (C.H.); s22020804046@cjlu.edu.cn (Q.Q.); p22020854015@cjlu.edu.cn (H.D.); mjg@cjlu.edu.cn (J.M.); wdh@cjlu.edu.cn (D.W.); s20020804040@cjlu.edu.cn (L.M.); msxu@cjlu.edu.cn (M.X.); 2 Zhejiang Engineering Research Center of Fluid Equipment and Measurement and Control Technology, Hangzhou 310018, China; 3 College of Energy Engineering, Zhejiang University, Hangzhou 310027, China; 12127107@zju.edu.cn

**Keywords:** carbon nanotubes, blocky composites, frictional property, aggregation effect

## Abstract

A large amount of primary energy is lost due to friction, and the study of new additive materials to improve friction performance is in line with the concept of low carbon. Carbon nanotubes (CNTs) have advantages in drag reduction and wear resistance with their hollow structure and self-lubricating properties. This review investigated the mechanism of improving friction properties of blocky composites (including polymer, metal, and ceramic-based composites) with CNTs’ incorporation. The characteristic tubular structure and the carbon film make low wear rate and friction coefficient on the surface. In addition, the effect of CNTs’ aggregation and interfacial bond strength on the wear resistance was analyzed. Within an appropriate concentration range of CNTs, the blocky composites exhibit better wear resistance properties. Based on the differences in drag reduction and wear resistance in different materials and preparation methods, further research directions of CNTs have been suggested.

## 1. Introduction

The phenomenon of friction is ubiquitous in life. Any process that requires force to change the state of motion of an object produces frictional resistance. Frictional resistance is a force that impedes the relative movement of objects. With the help of friction, we can move forward and car wheels can roll forward. At the same time, friction also accelerates the irreversible wear and tear of the contact surfaces; severe wear and tear even destroys the normal working state of the organism [1]; for example, cavitation phenomenon caused by fluid friction on the surface of flow blades [2,3]. The presence of friction gives way to the consumption of energy and leads to irreversible changes in the surface of the friction pair. Therefore, friction is usually considered as a negative effect in terms of industrial production [4,5]. A lot of primary energy is lost annually worldwide, and most machine components break due to friction or corrosion [6,7].

Currently, countries around the world are highly concerned about carbon emissions. How to improve the wear resistance of objects and achieve the purpose of being green and environmentally friendly has been the focus of tribology [8,9,10]. Composite materials break through the performance limitations of a single material and have better comprehensive performance [11]. Improving the friction characteristics of this new material has innovative significance [12,13], and high-strength and high-modulus nanomaterials provide ideas. Through in-depth research on carbon nanomaterials, it has been found that they have good corrosion resistance, excellent mechanical properties, etc. [14,15,16]. They are the strongest carbon fibers known to mankind with particular characteristics. Among them, the unique cylindrical hollow structure of carbon nanotubes (CNTs) [17], as well as self-lubrication [18], stand out among the wear-resistant lubrication additive materials and are considered to be the most suitable additive materials for reinforcement, toughening, and drag reduction. Chemical vapor deposition, as the most common and economical method for generating CNTs, decomposes carbon source gas under high temperature and catalyst action, rearranging carbon atoms to generate CNTs.

Based on elaborating on the interaction between CNTs and blocky composites, this paper analyzes the materials’ friction properties improvement by CNTs. It is an additive in three types of blocky composites (including polymers, ceramics, and metal matrix composites). This paper reviews the mechanism of CNTs as a solid lubricant, reveals the unique aggregation effect of CNTs in the blocky composites, analyzes friction properties (friction coefficients and wear rates) in terms of factors such as the preparation method, test conditions and sliding conditions, and finally, looks ahead to the challenges and research directions for CNTs. Related reviews have focused on the use of CNTs as lubricants and coatings to improve the frictional properties [5,19,20,21], and this article focuses on analyzing the blocky composites’ friction characteristics with CNTs.

## 2. The Interaction between CNTs and Blocky Composite Materials

Blocky composite material is a structural material composed of two or more different materials, each of which is combined in a block form and fixed together through bonding, welding, pressing, and other methods to form a complete structure. The tribology of blocky composites focuses on the relative motion of surfaces, the coefficient of friction (COF) of composites in a friction system, and the wear rate (WR). The improvement of the frictional properties is a direct way to reduce wear and energy consumption.

Currently, the improvement of friction properties of blocky composites is mainly through fiber reinforcement, the addition of solid lubricants, and the addition of metals and metal fillers. Fiber reinforcement refers to adding fibrous materials that increase the strength, stiffness, and wear resistance [22,23,24], such as carbon fibers, glass fibers, etc. Fibers can prevent wear and fracture of the material surface during friction, thus improving the durability and friction properties of the material [25]. However, the use of fiber reinforcement increases the cost as well as the manufacturing difficulty and process complexity. Adding metals or metallic fillers such as copper powder, aluminum powder, etc., to the blocky composites can increase the surface’s hardness and wear resistance because metallic fillers can form a hard coating during friction, thus reducing the wear and COF [26]. However, filling too much metal and metallic fillers will increase the density, cost, and also reduce the bending and tensile properties. Solid lubricants such as graphite, CNTs, and polytetrafluoroethylene can form a lubricating film and reduce the contact stress on the surface. It reduces the frictional resistance and improves the durability of the material. The addition of solid lubricants is less costly compared to fiber reinforcement and the addition of metals and metal fillers because solid lubricants are relatively inexpensive additives. Furthermore, the addition of solid lubricants can be more easily processed and fabricated so that the material surface becomes smoother.

**Figure 1:**
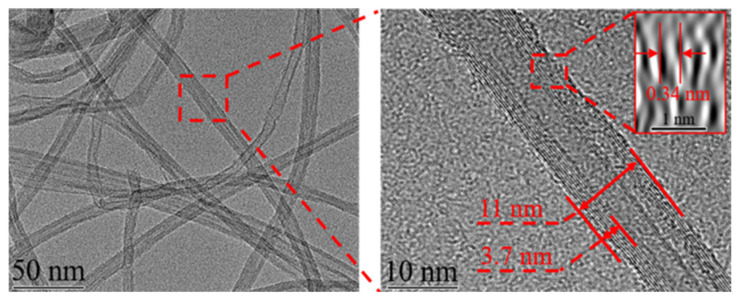
TEM observation of MWCNTs [29].

**Figure 2:**
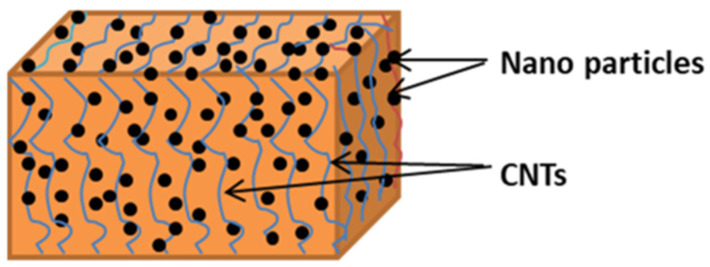
Schematic diagram of CNTs-reinforced nanocomposites [31].

CNTs, being a solid lubricant, have a unique tubular hollow structure, which can be viewed as hollow cylinders rolled into different lamellae of graphene. CNTs are categorized into single-walled CNTs (SWCNTs) and multi-walled CNTs (MWCNTs) according to the number of wall layers [27,28]. TEM observations of MWCNTs are shown in Figure 1. The MWCNTs are randomly distributed and entangled due to the van der Waals forces between the molecules. They exhibit one-dimensional tubular nanostructures of about 11 nm in diameter, with the hollow part about 4 nm in diameter and the thickness of the wall about 3.7 nm [29]. In addition, the stated size of nanotubes refers to their specific preparation. Generally, the size of MWCNTs can vary in a broad range [30]. The unique tubular hollow structure of CNTs provides the possibility to realize rolling friction and load dispersion on the composite surface. Figure 2 shows the schematic diagram of CNTs-reinforced composites. Due to sp2 hybridization where carbon atoms form covalent bonds with other atoms, van der Waals forces between molecules, hydrogen bonding, etc., the matrix molecules are tightly bound to CNT molecules, forming a composite material with excellent strength performance [31].

Blocky composites can be categorized as polymer-based, metal-based, and ceramic-based composites according to matrix materials. CNTs are effective as additives to improve the tribological properties of blocky composites [32]. Researchers are exploring their applications in chemical catalysis, energy conservation, and other fields [33]. However, while CNTs enhance the strength performance of blocky composite materials, there are also drawbacks such as poor dispersion and weak adhesion to the substrate surface which affect their level of enhancing wear-resistance properties of blocky composites.

## 3. Effect of CNTs on the Friction Properties of Polymer Materials

Due to the excellent properties, polymer materials can be seen everywhere [34,35]. To expand their applications in more fields, studies on enhancing the friction properties of polymer materials have been carried out [36]. Adding CNTs [37,38] or grafting CNTs [39] has been demonstrated to improve the tribological properties without affecting the adsorption of polymer molecules [40]. In the following, in addition to investigating the frictional properties’ improvement mechanism of polymer materials with CNTs and the unique aggregation effect, the results of preparation methods, test conditions, and sliding conditions on the frictional properties of CNTs containing polymer materials are investigated.

### 3.1. Improvement Mechanism

Adding CNTs improves the tribological properties of polymeric materials in three main ways:

1. Adding MWCNTs enhances the matrix material

The hardness of MWCNTs/ultra-high molecular-weight polyethylene (UHMWPE) composites prepared at different contents of MWCNTs showed that there was no significant change in hardness with the increasing content [40]. However, the wear deformation of the UHMWPE was reduced. Since the reduction in deformation was not related to the hardness, it was concluded that adding MWCNTs increased the shear strength of matrix material, which could effectively transfer the shear or applied loads to the polymer into the dispersed MWCNTs, resulting in a reduction in the amount of plastic deformation and the size of the separated particles [41].

2. Strong chemical covalent bonding between MWCNTs and substrate

**Figure 3:**
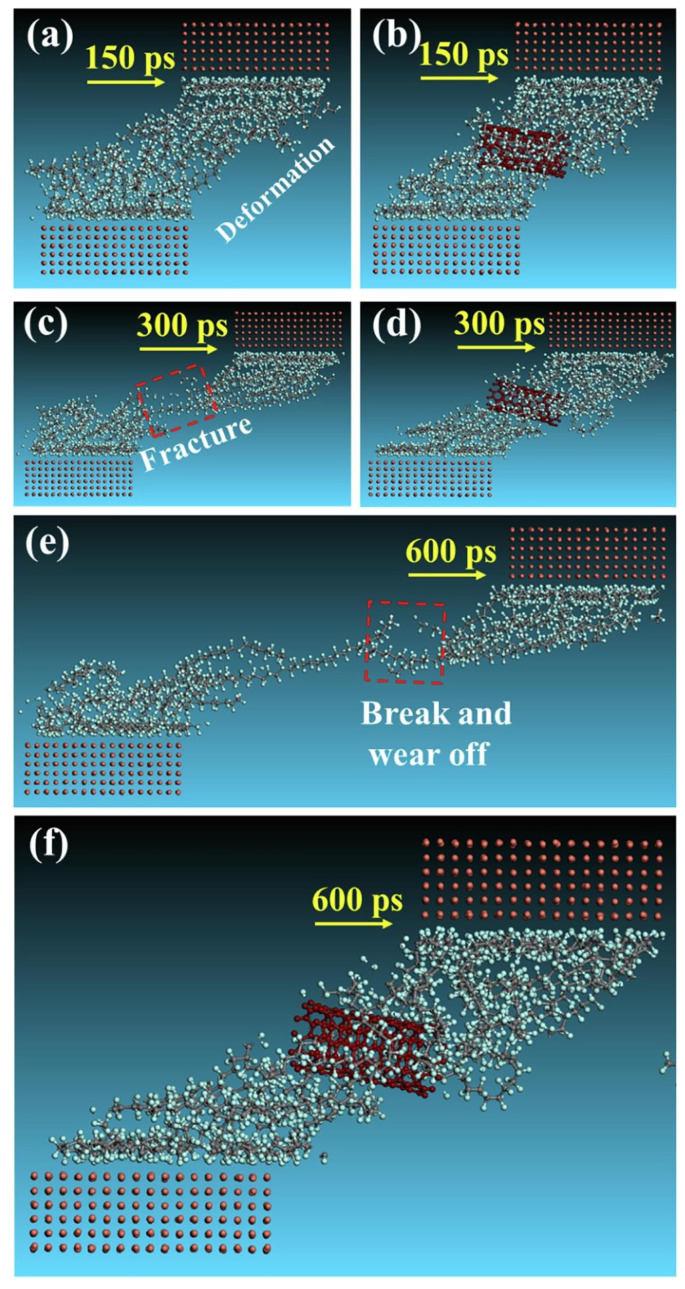
The snapshots of friction process of pure PTFE (**a**,**c**,**e**) and CNTs/PTFE composites (**b**,**d**,**f**) sliding against Cu layer at 150 ps, 300 ps, and 600 ps [42].

When exploring the tribological properties of MWCNTs-reinforced polytetrafluoroethylene (PTFE) materials through molecular dynamics (MD) simulations [42], the improvement effect of MWCNTs was due to limiting the shear and fracture during the contact period. The interaction between MWCNTs and PTFE molecules leads to MWCNTs having an adsorption effect on PTFE molecules, which makes PTFE reinforced and avoids its deformation and fracture. As shown in Figure 3, the wear mechanism is changed from viscous to abrasive wear, which contributes to the reduction of WR. Similar phenomena were found in molecular dynamics simulations of MWCNTs/nitrile butadiene rubber (NBR) composites when investigating different MWCNT modifications [43] and sliding velocities [44]. At the same time, adding MWCNTs did not affect the intermolecular adsorption of the matrix.

3. Formation of transfer films between CNTs and substrates

**Figure 4:**
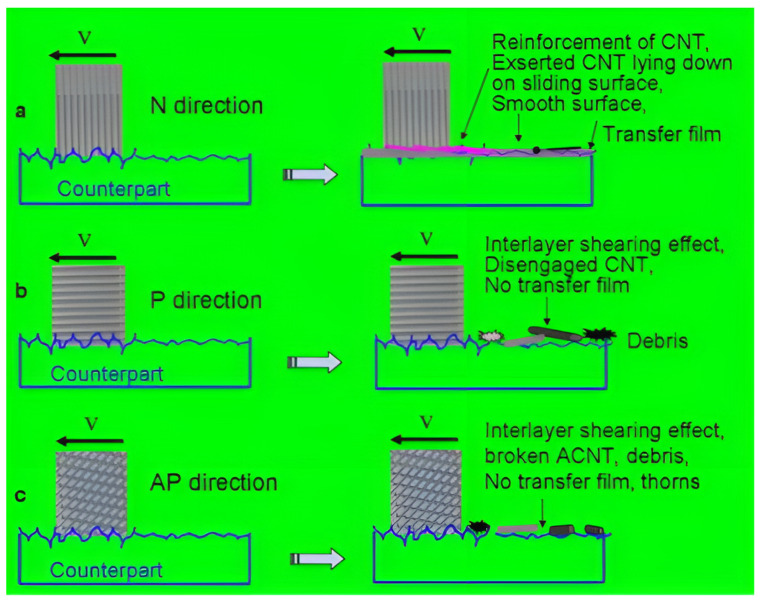
Mechanism of ACNTs’ orientation on the tribological properties of epoxy resin composites [47]. (**a**) Normal direction. (**b**) Parallel direction. (**c**) Antiparallel direction.

Due to the extremely high aspect ratio and anisotropic nature, the orientation of the CNTs in the polymer [45] and the transverse-to-longitudinal ratio [46] also affect the properties. Adding 1.1 vol% aligned CNTs (ACNTs) to the epoxy resin showed a superior improvement in friction properties compared to randomly dispersed CNTs. Among the three alignments, the WR in the normal direction is more improved than the parallel and perpendicular two [47]. Due to the weak bonding strength, it is easy to remove worn surfaces during friction. The fragments dislodged from ACNT in a normal direction can form a continuous transfer film, whereas the fragments dislodged when sliding in parallel or perpendicular directions are relatively large and cannot form a continuous transfer film during sliding from Figure 4.

**Figure 5:**
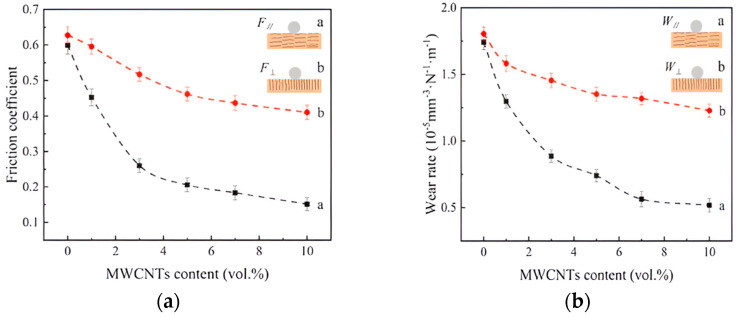
COF and WR of MWCNTs/Cu composites at different concentrations [49]. (**a**) Parallel to drawing direction. (**b**) Perpendicular to drawing direction. (In the figure, a refers to parallel direction, b refers to perpendicular direction).

The formation of transfer films is the reason why ACNTs improve the wear resistance of composites more effectively in the normal direction [48]. A similar situation was found in the enhancement of copper matrix composites using multi-walled CNTs [49], as shown in Figure 5, which exhibited different tribological properties’ improvement effects due to the different orientations of the CNTs, where the improvement effect in the parallel direction is significantly superior to the perpendicular direction, both in terms of the wear resistance and the reduction of COF.

### 3.2. Agglomeration

During the preparation of polymer composites, the structural integrity of the CNTs, the degree of dispersion in the polymer material, the degree of infiltration with the polymer and external factors (hygrothermal aging, γ-irradiation, etc.) [50] affect the composite properties with CNTs [51]. In particular, the aggregation of CNTs not only limits the application of CNTs but also weakens the improvement of tribological properties [52,53].

**Figure 6:**
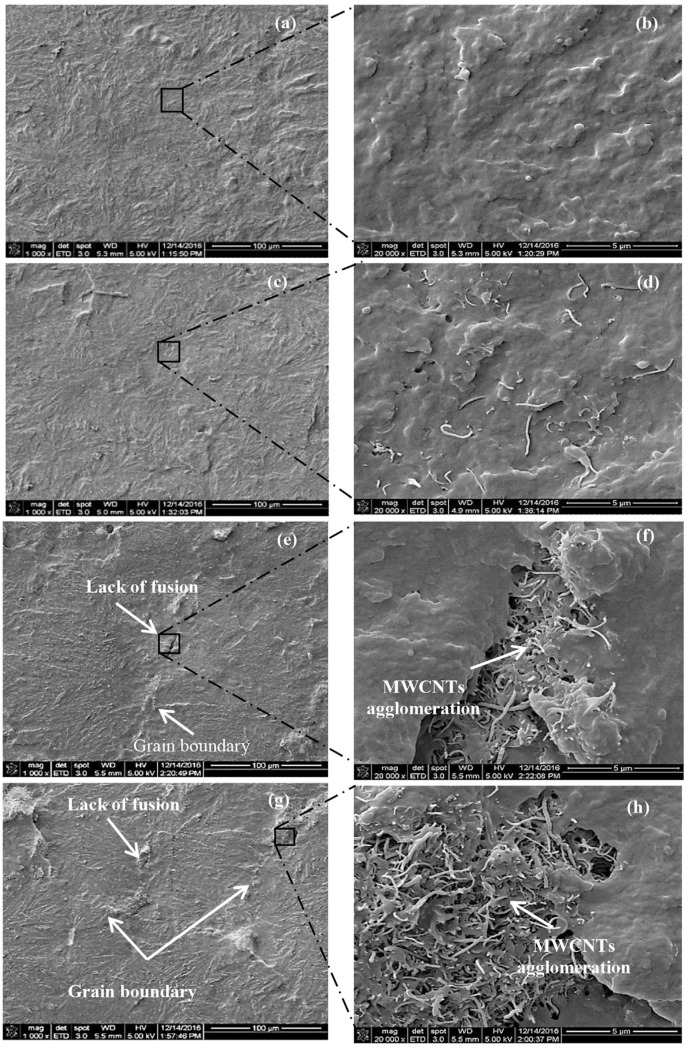
FE-SEM images of the transverse sections of consolidated samples at two different magnifications (1 K, left and 20 K, right). (**a**,**b**) UHMWPE GUR 1020; (**c**,**d**) Mixed 0.1wt% MWCNTs; (**e**,**f**) Mixed 0.5wt% MWCNTs; and (**g**,**h**) Mixed 1.0wt% MWCNTs [41].

CNTs’ aggregation effect in polymer matrices depends on two main aspects, i.e., the concentration and the dispersion method. On the one hand, for different polymers, CNTs are dispersed differently in them, and they also have different suitable concentrations. The improvement of the friction properties of polymers does not increase with the increase of concentration. When there is an excess of CNTs, the polymer composites lack fusion due to the aggregation effect of CNTs and produce high-density defects as illustrated in Figure 6. The presence of these defects will promote the generation of interlayer cracks and the propagation of fracture cracks, causing the production of large particles [41]. As the concentration of CNTs increases, aggregation will also become more pronounced, the degree of aggregation-induced defects will be enhanced, and the wear mechanism will be transformed into abrasive wear [40], deteriorating the frictional properties of the substrate material. A similar situation exists when adding excessive CNTs to Polyamide 6(PA6)/UHMWPE composites, where the adhesion between the excessive CNTs and the PA6 matrix becomes weaker, making the CNTs easy to peel off from the matrix to form wear debris [54]. Therefore, the selection of appropriate concentrations for different polymers is important on tribological properties.

On the other hand, the dispersion method, as one of the preparation processes of polymer composites, has a certain influence on the CNTs’ dispersion in the polymer. At present, CNTs’ dispersion in polymers mainly uses physical methods and chemical modification. Physical methods mainly include mechanical grinding, ultrasonication, and high-energy stirring, where mechanical grinding and ultrasonication techniques induce localized shear stresses to promote CNTs’ dispersion, while stirrers are generally used in conjunction with ultrasonication to aid in the mixing process. Chemical modification introduces functional groups and is a commonly used dispersion method for preparing polymer composites. Polymer chains containing aromatic rings are adsorbed on the sidewalls of CNTs (without destroying the structure of the CNTs) by van der Waals forces and π-π bonds. CNTs’ tendency of aggregating in the polymer matrix is weakened by mutual repulsion between functional groups [55].

**Figure 7:**
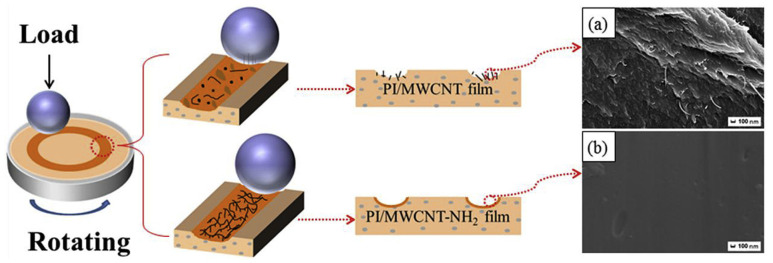
Friction mechanism model of PI/MWCNT and PI/MWCNT-NH_2_ films. SEM images of the wear surface of (**a**) PI/MWCNT-1wt% nanocomposite and (**b**) PI/MWCNT-NH_2_-1wt% nanocomposite [56].

MWCNT-NH_2_/polyimide (PI) nanocomposites were prepared using in situ polymerization with amine-functionalized MWCNTs, and the friction properties were improved compared to pure PI matrix materials and PI/MWCNT blends due to the uniform dispersion and the improved interfacial bonding [56]. As shown in Figure 7, with the same MWCNT concentration and friction conditions, MWCNT-NH_2_/polyimide (PI) nanocomposites have smoother surfaces and less wear. In addition, compared to MWCNTs/bismaleimide (BMI) obtained by utilizing the original treatment method, using atmospheric pressure filamentary dielectric barrier discharge (APDBP) resulted in enhanced MWCNT and BMI substrate infiltration and interfacial adhesion [57], with lower COF and WR. Therefore, improving the friction properties of polymer composites using different MWCNTs’ dispersion methods has different effects.

### 3.3. Preparation and Characterization

Various defects are inevitably generated during the preparation of CNTs, among which Stone-Wales (SV) and Vacancy (VA) defects are generally present [58]. The SV defect is a structural defect that occurs due to the rearrangement of carbon atoms and the VA defect arises from the absence of a carbon atom in the carbon nanotube, creating a vacancy. So complete CNTs exhibit the best COF and WR compared with SV and VA CNTs, therefore choosing a suitable preparation method can better utilize the friction properties of CNT polymer materials.

**Figure 8:**
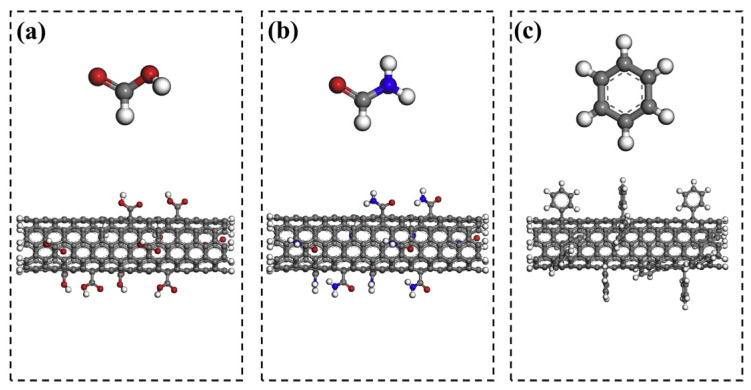
Snapshots of functionalized CNTs. (**a**) Carboxyl groups; (**b**) Amine groups; (**c**) Phenyl groups [59].

In situ polymerization and conventional metallurgy are common methods for preparing polymer composites. In situ polymerization is usually used to deal with insoluble and thermally unstable polymers that cannot be mixed by solution and have superior performance in interfacial bonding. The modification of CNTs by introducing functional groups can enhance the friction properties of composites. Three common functionalized functional groups are carboxyl, amine, and phenyl [59,60], as shown in Figure 8. The introduction of carboxyl groups improves the dispersion state; the introduction of amine groups improves the dispersion and reactivity of CNTs and can be carried out by simple laboratory setups without the need for complex equipment; the introduction of phenyl groups, which can be viewed as a substitution or addition reaction, avoids the occurrence of SV and VA defects. In addition to this, some molecules such as PDA [61] can also be used as access points for CNTs and polymers, thus improving the agglomeration effect of CNTs, and then the composites are prepared by the self-polymerization behavior of the molecules. However, excessive modification will destroy the graphite structure of CNTs and weaken their intrinsic mechanical properties. Therefore, optimization issues need to be considered for the modification methods and the degree of modification [62].

**Figure 9:**
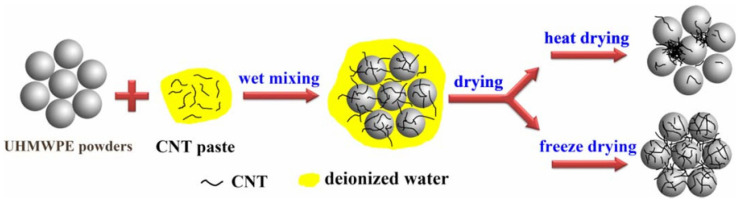
Effect of different drying methods on the dispersion state of CNTs [63].

In addition, regarding the molding of polymer blend powders, there are freeze-drying, heat-drying [63], and conventional metallurgy [41], with different preparation methods having different characteristics. Freeze-drying has superior performance for small amounts of CNTs, and its operation is simple and environmentally friendly [63]; thermal drying has similar characteristics to freeze-drying and has simpler and less cost, but the dispersion state of CNTs is worse than that of freeze-drying as shown in Figure 9; conventional metallurgy, which mainly includes hot pressing, cold isostatic pressing (CIP) [64], and the calendering process, has universal applicability and industrial application, but it cannot ensure the structural integrity of the CNTs in the preparation process.

Table 1 summarizes the effect of CNTs on the friction properties of different polymeric materials; CNTs’ concentrations and test conditions involved are the best parameters. As can be seen from Table 1, the addition of CNTs improves the friction properties of polymer materials to varying degrees, which proves the promising application of CNTs in the friction field [65]. With the same matrix material, CNTs’ concentration, test conditions, and preparation method, the composites show differences in lubrication conditions. Wet lubrication has better friction behavior compared to dry lubrication conditions, which is common sense. Different materials, different CNT contents, different lubrication conditions, different preparation methods, and different test conditions affect the friction behavior of the composites.

Meanwhile, as can be seen from Table 1, the optimal concentrations corresponding to different matrix materials are different. The mass fractions of the added CNTs are all within 1wt%, confirming the above statement that when the concentration of CNTs exceeds a certain limit, the frictional properties do not improve with the increase of CNTs due to the aggregation effect. Lower concentration of CNTs avoids the influence on other properties of the matrix material on the one hand and the deterioration of friction performance caused by CNTs’ aggregation on the other hand.

## 4. Effect of CNTs on the Friction Properties of Metal Matrix Composite Materials

Composites with metals and their alloys as the main components are known as metal matrix composites (MMCs). The metal substrate provides the stiffness or ductility of MMCs, and the addition of fillers further enhances the wear resistance and elastic modulus of MMCs, which is currently a widely studied topic. If macromaterials are used as reinforcements for metal matrix composites, the impact resistance and strength of the materials will be reduced, and the machinability of the materials will be adversely affected [67]. Whereas, the incorporation of high-hardness nanoparticles into matrix alloys will inevitably accelerate the wear of the matrix alloys or reduce the machinability of the components. Therefore, it is of practical significance to explore suitable additive materials in further improving the frictional properties of MMCs. The following study investigates the factors and mechanisms of CNTs to improve metal matrix composites’ friction properties and explores preparation methods, test conditions, and sliding conditions on the friction characteristics of metal matrix composites.

### 4.1. Factors Affecting Friction Performance

Metal matrix composites have numerous excellent properties, among which low density, high strength, high stiffness, and electrical conductivity are widely recognized in the automotive and aircraft industries, but the lower wear resistance limits their use in certain scenarios. Two main aspects affect the frictional behavior: (1) mechanical and external physical factors such as sliding velocity, load [68], and current [69]; (2) the frictional properties of the metal matrix composites themselves. Among them, the second factor plays a dominant role. Selecting CNT materials with nanoscale, excellent modulus of elasticity, low density, high aspect ratio, and self-lubricating properties as reinforcement materials provides feasible ideas for researchers [70]. The incorporation of CNTs not only ensures that the other properties of the composites are not compromised but also improves the tribological properties [71].

To carry out the improvement of metal matrix composites using CNTs, the first consideration is the wettability and interfacial bonding strength of the additive with matrix material, and the second consideration is the added content. The wettability and interfacial bond strength depend not only on the additive itself but also on the method of preparing the material. Different preparation methods not only affect the dispersion and the interfacial bond strength but may also affect the generation of carbides (Al_4_C_3_) [72]. Carbide generation is then accompanied by a decrease in the amount of CNTs in the material, as well as having an impact on the bond strength, which has a negative effect on the expected improvement.

### 4.2. Improvement Mechanism

**Figure 10:**
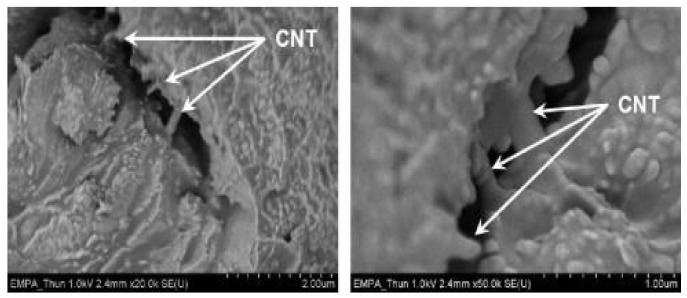
A SEM image of the fracture surface after tensile test (CNTs are pointed out with white arrows) [84]. (Scale bar: 2 μm on the left and 1 μm on the right).

Adding CNTs to improve friction properties in metal matrix composites often uses metals such as aluminum, magnesium, copper, and nickel as substrates [73,74]. However, aluminum matrix composites are most commonly used due to their wide applicability and low cost. During wear of aluminum matrix composites, both ambient and friction-generated temperatures are sufficient to oxidize the surface of aluminum matrix composites, contributing to an amorphous layer of aluminum oxide around the exposed aluminum particles and oxidative wear [75]. Aluminum oxides fracture and spall off during wear due to lower adhesion and harder strength than the aluminum matrix. As the aluminum matrix is gradually shed, CNTs are exposed to the matrix surface [76]. The occurrence of these processes improves frictional properties of the aluminum matrix composites in the following main ways:

1. CNTs exfoliated or exposed to the surface of the aluminum matrix are crushed or abraded [77] to form a thin carbon film [78]. Due to the self-lubricating properties, the carbon film has a low interfacial shear strength and can be used as a solid lubricant to reduce friction. Carbon is more easily oxidized than metal substrates [68] and can act as a barrier to oxidation reactions, where O^−^ and O^2−^ formed by friction processes will preferentially react with the filler. As a result, the combination of metal cations (Cu^+^, Cu^2+^, Al^3+^, etc.) with O^−^ and O^2−^ is delayed. The formation of carbon film inhibits oxidative and adhesive wear [79].
2. The carbon film reduces the area of direct contact with the substrate surface. When the content of CNTs is low, the carbon film cannot completely cover the wear surface, and with the increase of CNTs, the area of carbon film becomes larger gradually, which gradually reduces the area of direct contact, and the tribological performance is gradually improved.
3. Due to the high hardness and small radial size of the CNTs, after adding CNTs to the matrix, they roll between the surfaces of the friction pair [70,80], transforming sliding friction into rolling friction and resulting in the smallest wear [81], thus reducing the WR and COF of the material.
4. The incorporation of CNTs inhibited the grain growth, so the grain refinement further increased matrix hardness [82]. According to Archard’s law, the WR is reduced with the hardness [83], and an increase in hardness can improve wear resistance. As shown in Figure 10, some CNTs can be observed in the cracks, implying that CNTs have an inhibitory effect on crack propagation [84], and a similar phenomenon has been found in the friction performance study of Al6061-SiC-CNT [85].
5. CNTs reduce energy loss and heat generation while reducing friction, and good interfacial bonding with the substrate improves heat transfer efficiency and reduces temperature rise. In addition, good interfacial bonding can reduce oxidation and thermal corrosion on the metal surface, further reducing the temperature rise.

In addition, when CNTs are added above a certain content, the COF and WR of aluminum matrix composites will exhibit deteriorating effects if they continue to increase [86,87]. On the one hand, due to the aggregation of CNTs, there are aggregated CNTs in different parts of the matrix, which cannot provide a continuous carbon nanotube film to improve wall contact conditions during the friction process; on the other hand, the coalescence formed is subjected to concentrated stress, which leads to cracking of the material during friction, and the wear mechanism becomes a delamination of the wear, which produces more wear debris and deteriorates wear effect. At the same time, the hardness of the material deteriorates, which affects the wear resistance of CNTs.

### 4.3. Preparation and Characterization

Currently, the commonly used preparation methods for metal matrix composites containing CNTs include powder metallurgy [78], molecular-level mixing (MLM) [88], friction stir processing (FSP) [85], and in situ synthesis [89,90,91], etc. The different preparation methods have different characteristics.

**Figure 11:**
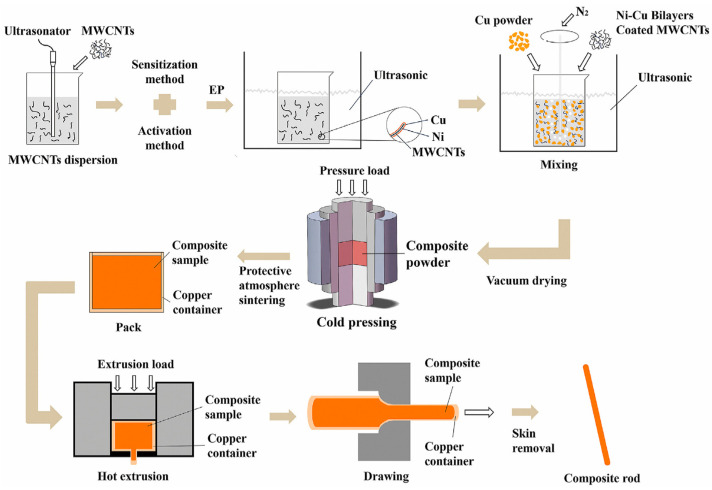
Powder metallurgy process [49].

**Figure 12:**
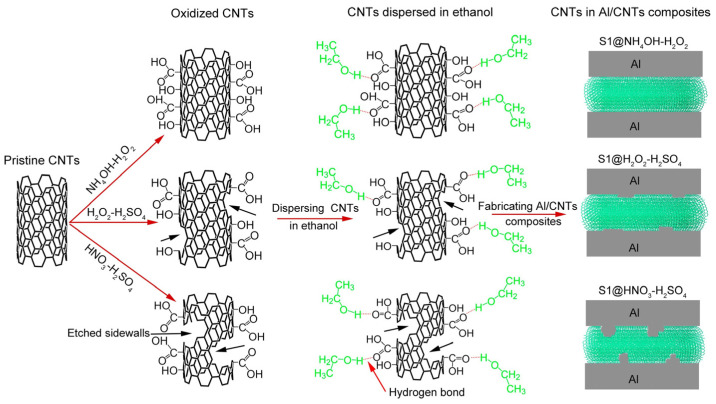
Modification results of CNTs after chemical oxidization and schematic diagram of the interfacial structure in Al/CNTs’ composites [97].

Powder metallurgy is a traditional composite material preparation method, as shown in Figure 11, which firstly makes CNTs well-dispersed in the composite material by using dispersion methods such as grinding dispersion [78,92], ultrasonic dispersion [72], stirring dispersion [93,94], and then the CNTs and the metal material are compressed and kneaded into a blocky material by using hot extrusion, vacuum sintering [69], cold pressing [49], pressureless infiltration [76], spark plasma sintering (SPS) [95], and equal channel angular pressing (ECAP) [96]. However, powder metallurgy cannot achieve direct interface bonding, and the interface bonding strength is weak [93], while the introduction of functional groups to pretreat CNTs [97] enhances the anchoring of CNTs in the matrix material, but the preparation process is complicated and cumbersome to control, which destroys the structure of CNTs, as shown in Figure 12. In addition, for the widely used planetary ball milling dispersion method in powder metallurgy, the prolonged grinding and deformation of metal powders may result in excessive cold welding, which ultimately leads to large particle sizes that inhibit the dispersion of CNTs. It has been shown that the repetitive deformation ball milling (RDBM) process reactivates the plastic deformation of cold-welded aluminum powders and refines the matrix grains to the nanoscale [98].

**Figure 13:**
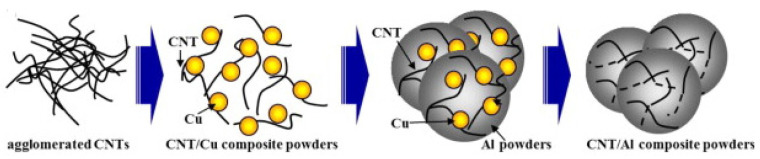
Preparation of CNT/Al-Cu composites by MLM [100].

The preparation of CNTs containing metal-based materials using MLM, as shown in Figure 13, can contribute well to the dispersion in metal-based materials and improve the interfacial bonding strength, but the process is complicated, and the cost is high, which does not have the prospect of industrialization [99].

**Figure 14:**
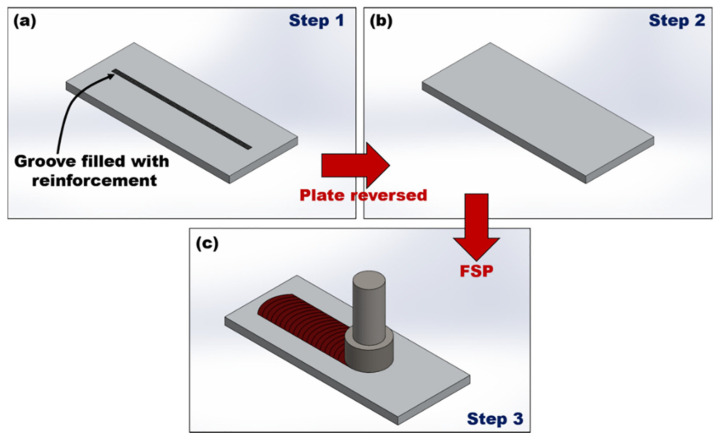
Schematic view of FSP [85]. (**a**) Plate with groove. (**b**) Plate reverse. (**c**) Stirred.

FSP is considered to be a solid-state plastic deformation method without fear of reaction between CNTs and molten metal, as shown in Figure 14. During the stirring process, the metal matrix material is forcibly stirred and forms a plastic deformation zone, resulting in grain refinement of the metal matrix. During this process, interface bonding occurs between CNTs and the metal matrix forming a strong interface. FSP avoids the low infiltration of CNTs in the metal matrix and carbides caused by the powder metallurgy method, but the high content of CNTs is prone to agglomeration and degradation [82], which limits the development of this preparation method [101].

**Figure 15:**
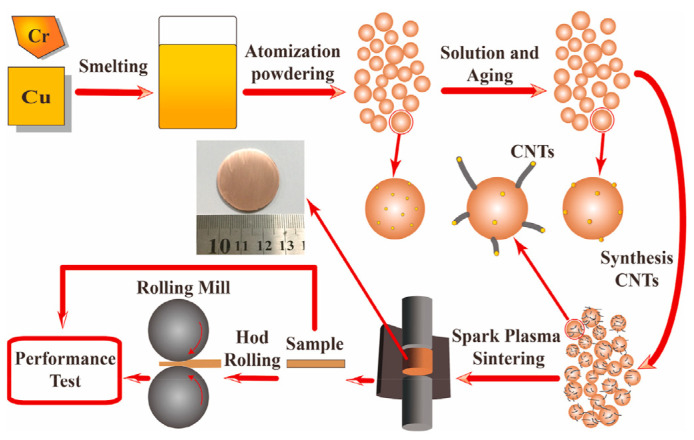
Preparation of CNTs/CuCrZrY composites by in situ synthesis [95].

As shown in Figure 15, in situ synthesis of CNTs/CuCrZrY composite powder using Cr particle catalysts uniformly distributed on the surface of CuCrZrY composite powder. Finally, the CNTs/CuCrZrY powders are cured by spark plasma sintering and compaction [95]. Although this method promotes good interfacial bond strength between CNTs and metal matrix, the dispersion state is related to the surface catalyst dispersion state, the reaction time needs to be controlled, and the preparation process is complicated.

Therefore, it is of positive significance for improving friction properties to choose suitable preparation methods according to different matrix materials to promote uniform dispersion and good interfacial bond strength between CNTs and metal-based materials. Table 2 summarizes the effect of CNTs on the friction properties of different metal matrix composite materials. The CNTs’ concentrations and test conditions involved are the best parameters.

As can be seen from Table 2, the addition of CNTs has produced certain improvement effects on the tribological properties of metal matrix composites, which proves the potential of CNTs as additives in the field of metal matrix materials’ tribology. However, the improvement effect for different metal matrix materials has a large difference, partly due to the different main improvement mechanisms of CNTs caused by the material itself and partly due to the different test conditions. The improvement mechanism of CNTs on other metal matrix composites is similar to the mechanism mentioned above on an aluminum metal matrix [102], and the main mechanism is that the incorporation of CNTs induces the formation of a carbon film during the wear process and the wear mechanism changes [103]. Meanwhile, the difference in preparation methods determines whether the CNTs can adhere well to the material and whether they can be uniformly dispersed in the substrate. Good interfacial bonding and homogeneous dispersion influence the effect of tribological behavior improvement [79].

**Table 2:** Frictional properties of CNTs in different metal matrix composite materials.

Material	Preparation Method	Characteristic	Sliding Conditions	Test Conditions	COF	COF Reduction Rate	Wear Reduction Rate	Source
0.8wt% CNTs/CuCrZrY	Water-assisted chemical vapor deposition (CVD) and SPS in situ synthesis.	Uniform dispersion and interfacial bonding; the process is reproducible.	Dry	10 N 200 rpm 5 min	0.254	38%	30%	[95]
10 vol% MWCNTs/Cu	Ultrasonic and nitrogen-assisted powder mixing, oriented and arranged in copper matrix by hot extrusion and cold drawing.	Enhance the homogeneous mixing and interfacial bonding strength of CNTs with Cu substrate.	Dry	5 N 50 mm/s 50 min	0.151	74.7%	70.3%	[49]
2wt% MWCNTs /Cu−10Sn	Traditional powder metallurgy route	CNTs were embedded inside the matrix and the powder particles underwent significant plastic deformation.	Dry	25 N 100 r/min 30 min	0.31	52%	60%	[103]
20vol% MWCNTs/Mg-Al	Pressureless infiltration process	Uniformly distributed; easy to control volume fraction; and no expensive equipment.	Dry	30 N 0.1571 m/s 25 min	0.105	28%	28%	[76]
10 vol % MWCNTs/Cu	The powders of copper and CNTs were mixed and milled. After mixing, the powder mixture was cold pressed and sintered.	Well-mixed and fully embedded in the matrix material, but mechanical milling disrupted the structure of the MWCNTs.	Dry	20 N 5 m/s —	0.11	42%	48%	[69]
15vol%MWCNT/Cu	CNTs were mixed with copper metal powder and sintered by microwave heating method.	Uniformly distributed, good interfacial bond strength, reduce the cost and ensure the integrity of the material.	Dry	12 N 2.77 m/s 12,330 m	0.09	50%	67%	[104]
CNTs-Al/Ni	Prepared by MLM, mixed with aluminum matrix powders by mechanical alloying method and cured by SPS.	Agglomeration of CNTs was prevented, a strong interfacial bond with the substrate, but a high cost.	Dry	20 N 1.1 m/s 25 min	0.578	18%	74%	[88]
0.5wt% CNTs/AZ31 (as cast)	Stir-casting method	Well-dispersed in the matrix and has good wettability with metals.	R68 oil	50 N 140 r/min 10 min	0.065	30%	5%	[105]

## 5. Effect of CNTs on the Friction Properties of Ceramic Matrix Composite Materials

**Figure 16:**
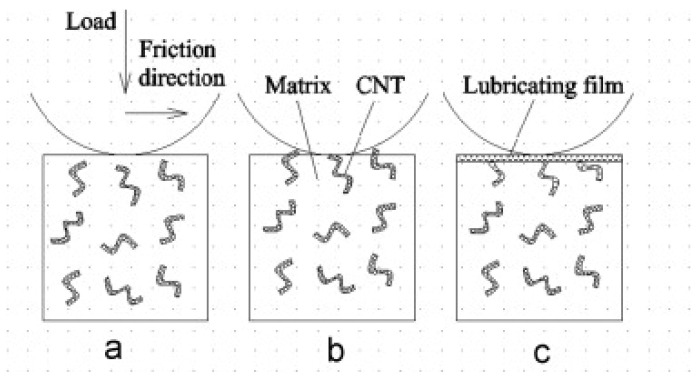
Schematic diagram of the formation process for Al_2_O_3_-CNTs composites’ self-lubricating film [107]. (**a**) Load pressure. (**b**) Friction process. (**c**) Formation of lubricating film.

Ceramic matrix composites (CMCs) have strong hardness and wear-resistant properties. However, they are brittle and highly susceptible to fracture. The current research on CNTs as additives is mainly on toughening ceramics and less in-depth research on the tribological properties of ceramics. The addition of CNTs, which produces a graphite layer during the friction process and forms the so-called transfer film, as shown in Figure 16, will undeniably improve ceramics’ friction properties. It is the ideal nanomaterial additive to enhance the wear resistance and lubricity of ceramic materials [106,107].

**Figure 17:**
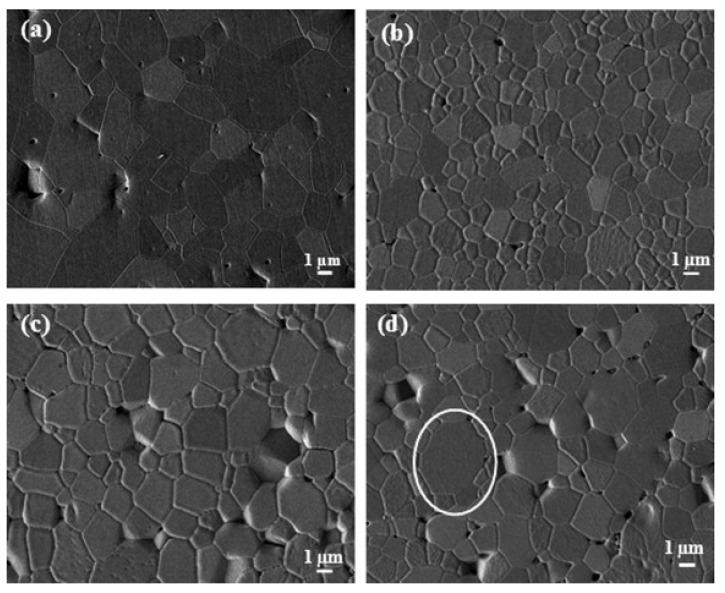
SEM micrographs of polished and thermally etched (**a**) pure Al_2_O_3_; (**b**) Al_2_O_3_ + 0.1wt% MWCNTs; (**c**) Al_2_O_3_ + 0.1wt% SWCNTs; (**d**) Al_2_O_3_ + 0.05wt% MWCNTs + 0.05wt% SWCNTs. Oval markers in the figure are the resulting coarse-grained microstructures [109]. (the white circle means coarse-grained microstructures).

When adding CNTs to ceramic matrix composites, MWCNTs are generally chosen as additives. The reasons for this are that MWCNTs are easier to disperse uniformly compared to SWCNTs and MWCNT/SWCNT blends, and MWCNTs’ low costing. In addition, in alumina ceramic materials, the incorporation of MWCNTs suppressed grain growth compared to the other two, forming a very fine nanograin structure as shown in Figure 17. The other two increased the mobility of grain boundaries during sintering, leading to some coarse-grained microstructures. The formation of coarse-grained structures adversely affects fracture resistance. The addition of MWCNTs increased composite materials’ hardness [108] and showed better friction property improvement compared to the other two [109].

**Figure 18:**
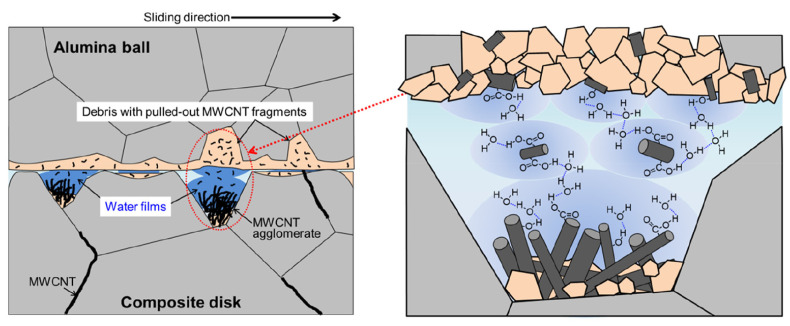
Schematic diagram of the formation of hydrodynamic lubrication film [110].

As shown in Figure 18, if the acidified MWCNTs are selected to improve the friction behavior of alumina ceramics, the functional groups adsorbed on the MWCNT fragments not only improve the agglomeration effect of CNTs but also participate in the hydrogen bonding network of the hydrodynamic lubrication film (water film), which accelerates the transition of the lubrication mechanism. The COF is 32–37% lower than that of alumina ceramic materials and comparative MWCNTs’ composites [110].

The unique aggregation effect of CNTs also applies to ceramic matrix composites, and its effectiveness in improving friction behavior also tends to increase and then decrease with the increase of CNTs’ concentration [111]. However, ceramic matrix composites can accommodate a higher volume fraction of CNTs than metal matrix composites and polymeric materials [31]. This is because the hydrophilicity of the substrate influences the amount of reinforcement that can be mixed, and ceramics are more hydrophilic than metals and polymers, allowing for the mixing of higher concentrations of CNTs. For the CNTs’ dispersion, the commonly used methods for surface functionalization are powder processing, sol-gel [112], and colloidal processing [113].

**Figure 19:**
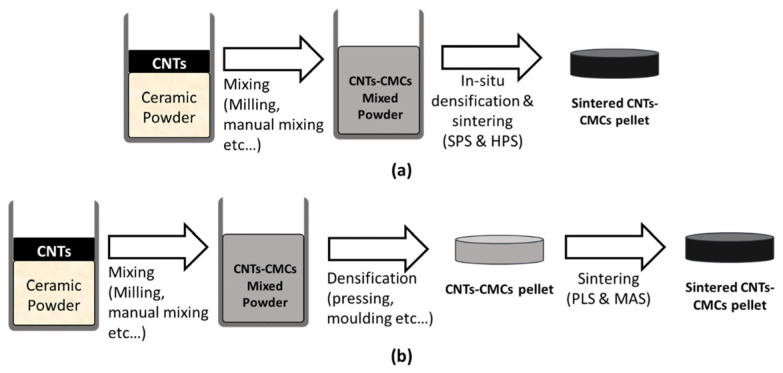
Two-step and three-step powder processing [114]. (**a**) Two-step; (**b**) Three-step.

It is common to prepare CNT ceramic matrix composites through powder processing, which typically requires two to three steps [114]. Figure 19 compares two-step and three-step powder processing. Both adopt the process of material mixing, densification, and sintering, but the difference is that the densification and sintering methods are different, such as in situ densification and spark plasma sintering for two-step processing, pressureless sintering, and microwave-assisted sintering for three-step processing. The mixing process is often carried out using dry CNTs and ceramic powders, with wet mixing using a solvent such as alcohol or acetone, followed by evaporation of the solvent. When preparing CMC with a large admixture of CNTs, it has the lowest complexity and operating cost compared to other fabrication techniques.

Colloidal processing involves a variety of physical/chemical methods to disperse CNT with water/solvents, employing dispersion steps such as ultrasonication, magnetic stirring, and chemical oxidation to convert it into CNT nanofluids, which in turn are added to ceramic powders and densified to obtain ceramic matrix composite products. Whereas the choice of CNT dispersant is limited [114] as it needs to fulfill the following sintering steps.

The sol-gel treatment method provides another method for forming dispersed CNTs in inorganic matrices [115]. However, the sintering process leads to partial deglassing of the silica matrix, which can result in a non-uniform microstructure [116]. The use of organic silane for surface modification can improve the aggregation problem of CNTs in precursor suspensions, achieving effective dispersion of silicate matrix at high concentrations of 3wt% CNTs [117].

Molding is the key to the manufacture of ceramic composites, and it largely determines the properties of ceramic composites. There is a wide variety of current molding methods, none of which is optimal in all possible ways, and there are always specific advantages and disadvantages. The current molding of CNTs’ hybrid ceramic matrix composites is usually made by sintering under vacuum or inert gas protection to prevent the CNTs from being destroyed by oxidation during the sintering process [118]. Conventionally, fine-grained and high-density CMCs reinforced with CNTs can be prepared by high-pressure or high-heating-rate sintering, such as hot pressing and pulsed plasma sintering [119]. However, these methods require expensive and complicated instruments. CNT/zinc silicate (CNT/ZS) composites prepared by a combination of powder processing and pressureless sintering are simpler and less costly, providing a new idea for preparing CNT/ZS composites [120].

As can be seen from Table 3, after adding CNTs, the friction characteristics of ceramic matrix composites are similar to those of polymer and metal matrix composites. Within an appropriate concentration range, the friction characteristics of ceramic matrix composites increase with the increase of CNTs’ concentration. In addition, the preparation method and testing condition also have a certain influence on the friction characteristics.

## 6. Summary and Outlook

The friction and wear phenomena that can be seen everywhere cause a large waste of primary energy, and the selection of suitable wear-resistant additive materials to change wear-resistant properties of the blocky composites is a proven energy-saving method, so the development of low COF and low wear reduction rates of the additive materials has a wide range of significance. The incorporation of CNTs into blocky composites, due to their characteristic tubular structure and the carbon film, effectively enhances the mechanical strength of the composites and reduces the amount of wear in the friction process of the material, which attracts a lot of research interests.

The blocky composites’ tribological properties with CNTs mainly depends on the dispersion of CNTs in the matrix and the strength of bonding with the interface. Good dispersion and strength of interfacial bonding cause the improvement of frictional properties. These two factors mainly depend on the CNTs’ orientation, CNTs’ concentration, matrix material, and preparation method. The article summarizes and analyzes the current universal dispersion, modification, and development methods of CNTs added to polymer materials, metal-based materials, and ceramic-based materials and provides basic theoretical guidance for the future tribology development of CNTs.

However, the current dispersion of CNTs in blocky composites still has much room for improvement, which has also somewhat weakened the display of good drag reduction and wear resistance. Therefore, it is necessary to focus on the homogeneous dispersion of CNTs in future research and further improve the preparation or modification method to extend CNTs’ dispersion performance under the premise of ensuring the structural integrity of CNTs. In addition, the current study also lacks an accurate model of CNTs’ dispersion state in matrix materials. In the future, a dispersion characterization model can be established by combining the experimental data with the molecular-level simulation so as to better investigate the mechanistic changes of CNTs’ antiwear properties under different dispersion states.

To solve the weak interfacial bonding strength between the CNTs and matrix, new additives can be developed from the hybridization perspective to help CNTs and matrix materials to better organic fusion. By relying on the intermediate medium to enhance the interfacial bonding strength, the duration of composite materials’ wear resistance can be improved. In addition, modeling of the interfacial bonding can be considered to compare the experimental results with the model calculations, and it can be further predicted in the future for experimental optimization studies.

## Figures and Tables

**Table 1:** Frictional properties of CNTs in different polymer materials.

Material	Preparation Method	Test Conditions	Sliding Conditions	COF	COF Reduction Rate	WR	Wear Reduction Rate	Source
0.5wt% MWCNT/UHMWPE (ultra-high molecular-weight polyethylene)	Ultrasonic agitation/hot press	6.5 N 60 mm/s 1680 m	Dry	0.15	18%	4.865 × 10^−6^ mm^3^/N·m	32%	[41]
0.5wt% MWCNT/UHMWPE	Ultrasonic agitation/hot press	6.5 N 60 mm/s 1680 m	Distilled water	0.1	10%	4.492 × 10^−6^ mm^3^/N·m	21%	[41]
0.7wt% MWCNT-NH_2_/PI	Introducing amide groups/in situ polymerization	5 N 300 r/min 170 m	Dry	0.31	25.2%	2.235 × 10^−4^ mm^3^/N·m	73.7%	[56]
0.7wt% MWCNTs-COOH/PI	Introducing carboxyl groups/in situ polymerization	3 N 0.157 m/s 282 m	Seawater	0.17	40%	5 × 10^−5^ mm^3^/N·m	76%	[52]
0.5wt% MWCNTs/epoxy	Mechanical dispersion/calendering process	10 N 0.09 m/s 1000 m	Dry	0.06	87%	3 × 10^−4^ mm^3^/N·m	93%	[66]
0.1wt% MWCNT/UHMWPE	Ultrasonic dispersion/simple mixing	5 N 200 rpm 2 h	Dry	0.1	71%	9.46 × 10^−5^ mm^3^/N·m	84.2%	[46]
1wt% MWCNTs/UHMWPE	Mechanical dispersion/freeze-drying	20 N 0.2 m/s 2 h	Dry	0.09	0%	3.15 × 10^−6^ mm^3^/N·m	28%	[63]
CNTs–PDA/PU_3D_/EP	PDA modification/in situ polymerization	1 Mpa 0.51 m/s 40 min	Dry	0.54	3%	1 × 10^−4^ mm^3^/N·m	61%	[61]

**Table 3:** Frictional properties of CNTs in different ceramic matrix composites.

Material	Preparation Method	Test Conditions	Sliding Conditions	COF	COF Reduction Rate	Source
10wt% CNTs/Al_2_O_3_	SPS	5 N 15 cm/s 100 m	Dry	0.22	66%	[106]
5wt% MWCNTs/Al_2_O_3_	SPS	50 N 100 °C	Dry	0.36	40%	[107]
10wt% MWCNTs/Al_2_O_3_	SPS	50 N 100 °C	Dry	0.25	58%	[107]
10wt% acid-treated MWCNTs/Al_2_O_3_	Acid-treated/mechanical mixing	1 N 0.1 m/s 10,000 m	Water	0.06	67%	[110]

## Data Availability

Not applicable.
